# Appointment Wait Times for Specialty Care in Veterans Health Administration Facilities vs Community Medical Centers

**DOI:** 10.1001/jamanetworkopen.2020.14313

**Published:** 2020-08-26

**Authors:** Kevin N. Griffith, Nambi J. Ndugga, Steven D. Pizer

**Affiliations:** 1 Department of Health Law, Policy and Management, Boston University School of Public Health, Boston, Massachusetts; 2 Partnered Evidence-Based Policy Resource Center, VA Boston Healthcare System, Boston, Massachusetts

## Abstract

This cross-sectional study compares appointment wait times at Veterans Health Administration facilities and those at community medical centers accessed via the Veterans Choice Program.

## Introduction

In 2014, the US Congress authorized the Veterans Choice Program (VCP), which allowed veterans to access care in the community if they lived more than 40 miles from the closest Veterans Health Administration (VHA) medical facility, could not schedule an appointment within 30 days or by the date clinically necessary (whichever was sooner), or experienced other specific hardships.^1,2^ Since the VCP’s rollout in the fall of 2014, approximately 2 million veterans have used the program.^3^ In this study, our objectives were to determine whether VHA wait times declined after VCP implementation, compare appointment wait times for specialty care in VHA vs community medical centers, and identify the proportions of community care wait times that were attributable to VHA administrative processes.

## Methods

This cross-sectional study was approved by the VA Boston Healthcare System’s institutional review board and adheres to the Strengthening the Reporting of Observational Studies in Epidemiology (STROBE) reporting guideline for cross-sectional studies. Informed consent was waived per institutional policy because the research, which includes millions of veterans, could not be practicably carried out without the waiver or an alteration of the study. 

We queried VHA administrative data to identify new consultation requests in 4 medical specialties: cardiology, gastroenterology, orthopedics, and urology. These were selected because they are high-volume purchased services under VCP and include surgical and nonsurgical specialties. We calculated mean appointment wait times for VHA care from 2013 to 2019, identifying changes in access after VCP implementation. We then compared wait times for VHA and community care at the medical center level in the 2018 to 2019 period. Because referrals to community care require confirmation of eligibility, we calculated the proportions of community care wait times that were attributable to VHA approval processes. More details about study methods are provided in the eAppendix in the Supplement. Analyses were conducted from September 2019 through February 2020 using R statistical software version 3.5.3 (R Project for Statistical Computing).

## Results

The VHA completed 6.9 million consultations in house and paid for an additional 869 000 consultations in the community for cardiology, gastroenterology, orthopedics, and urology from January 2013 to December 2019. The study sample consisted of 3 135 530 veterans, with mean (SD) age 62.0 (13.4) years; 2 873 521 (91.6%) were men. Mean wait times for VHA appointments declined during this period (Figure), but the declines began before VCP implementation. Orthopedics experienced the largest decline in mean (SD) wait times, from 53.0 (22.4) days to 30.0 (14.7) days. Lesser declines were observed for urology (42.0 [18.5] days vs 34.0 [11.7] days) and gastroenterology (58.0 [23.3] days vs 51.0 [22.9] days). Cardiology wait times did not differ over time.

**Figure.**
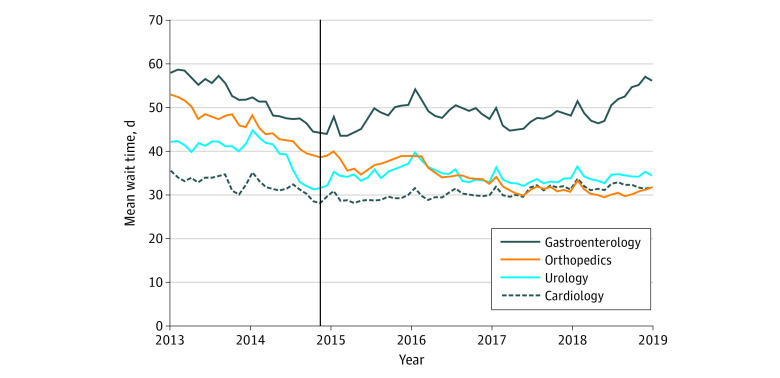
Appointment Wait Times for Specialty Care Within the Veterans Health Administration From 2013 to 2019 The Department of Veterans Affairs issued interim final rules implementing the Veterans Choice Program on November 5, 2014, indicated by the vertical black line.

VHA authorizations for community care added a mean (SD) of 6.4 (5.1) days to wait times for these appointments (Table). Even after excluding administrative delays (ie, the time it took for the local VHA to approve referral to community care), VHA facilities generally had lower wait times vs community facilities. Compared with community facilities, wait times were lower at 72 of 126 VHA centers for cardiology consultations (57.1%) (with 5 043 778 of 8 070 781 veteran enrollees who were assigned to centers in the cardiology group [62.5%] served at these lower-wait centers), 59 of 121 VHA centers for gastroenterology (48.8%) (serving 3 603 070 of 7 927 198 enrollees [45.5%]), 65 of 103 VHA centers for orthopedics (63.1%) (serving 4 528 072 of 7 817 934 enrollees [57.9%]), 49 of 75 VHA centers for urology (65.3%) (serving 3 985 400 of 7 944 144 enrollees [50.2%]), and 75 of 129 VHA centers overall (58.1%) (serving 4 876 861 of 8 138 943 enrollees [59.9%]).

**Table.** VHA Specialty Care Referrals 2018-2019, by Site of Care

Specialty	VHA		Community care			Percentage with lower VHA wait times^c^	
	Consultations, No.	Wait time, mean (SD), d	Consultations, No.	Administrative delay, mean (SD), d^a^	Wait time, mean (SD), d^b^	Medical centers	Enrollees
Cardiology	2 282 328	33.0 (8.7)	263 177	4.5 (2.9)	38.0 (9.2)	57.1	62.5
Gastroenterology	2 322 142	53.9 (15.9)	349 644	7.9 (6.9)	60.3 (16.0)	48.8	45.5
Orthopedics	1 364 562	36.2 (9.3)	206 644	6.5 (3.7)	43.6 (12.9)	63.1	57.9
Urology	960 314	36.1 (9.5)	49 381	5.8 (4.7)	50.5 (14.5)	65.3	50.2
Overall	6 929 346	41.1 (15.9)	868 846	6.4 (5.1)	49.0 (15.5)	58.1	59.9

Abbreviation: VHA, Veterans Health Administration.

^a^ The date difference between when the patient was initially referred to community care and when the request was approved by the local VHA medical center.

^b^ Excludes administrative delays.

^c^ Columns indicate what percentage of VHA medical centers offered lower wait times compared with community care options for a given specialty and what percentage of veteran enrollees were assigned to centers with lower wait times for a given specialty.

The mean (SD) wait times at VHA centers vs community facilities (excluding administrative delays for community facilities) were 33.0 (8.7) days vs 38.0 (9.2) days for cardiology, 53.9 (15.9) days vs 60.3 (16.0) days for gastroenterology, 36.2 (9.3) days vs 43.6 (12.9) days for orthopedics, 36.1 (9.5) days vs 50.5 (14.5) days for urology, and 41.1 (15.9) days vs 49.0 (15.5) days overall. Total wait times for VHA and community care were positively correlated; the weighted Pearson correlation coefficients between wait times were strongest for orthopedics (*r* = 0.50), followed by gastroenterology (*r* = 0.48). Cardiology (*r* = 0.21) and urology (*r* = 0.30) showed weaker correlations.

## Discussion

We found that, in general, VHA wait times declined between January 2013 and December 2019. Mean wait times were lower at most VHA medical centers compared with community care alternatives, even after accounting for administrative delays. These findings comport with previous research.^4^^,^^5^ Areas with higher VHA wait times frequently had longer wait times for community care as well.

There are limitations to our study. A portion of VHA referrals to community care may be misclassified as in-house consultations, and specialty is not recorded for some community referrals (eAppendix in the Supplement). Additionally, we cannot identify consultation urgency or conclude from these data whether the wait times we found are clinically appropriate.

In summary, VCP implementation in 2014 was not associated with lower specialty care wait times. Our findings suggest that policies that liberalize veterans’ eligibility for community care may be insufficient to lower veterans’ wait times in underserved areas.

## Notes

Supplement. **eAppendix.** Supplementary Methods
