# Error in Table

**DOI:** 10.1001/jamanetworkopen.2022.0642

**Published:** 2022-02-07

In the Research Letter titled “Appointment Wait Times for Specialty Care in Veterans Health Administration Facilities vs Community Medical Centers,”^1^ published August 26, 2020, there were errors in the number of consultations listed in the Table. This article has been corrected.^1^
