# Supplementary material for: Appointment Wait Times for Specialty Care in Veterans Health Administration Facilities vs Community Medical Centers
Source: JAMA Netw Open. 2020 Aug 26;3(8):e2014313. doi: 10.1001/jamanetworkopen.2020.14313 (PMC7450350; doi:10.1001/jamanetworkopen.2020.14313)
Supplement: Supplement. — eAppendix. Supplementary Methods [file jamanetwopen-3-e2014313-s001.pdf]

Supplementary Online Content

Griffith KN, Ndugga NJ, Pizer SD. Appointment wait times for specialty care in Veterans Health Administration facilities vs community medical centers. *JAMA Netw Open*. 2020;3(8):e2014313. doi:10.1001/jamanetworkopen.2020.14313

eAppendix. Supplementary Methods

This supplementary material has been provided by the authors to give readers additional information about their work.

© 2020 Griffith KN et al. *JAMA Network Open.*

eAppendix. Supplementary Methods

In this study, we extracted data from the Veterans Health Administration (VHA) Corporate Data Warehouse (CDW) to calculate consult wait times for both VHA and non-VHA community care (CC). The latest version of our analytic code is available at http://kevin-griffith.com/resources/.

Only consults with a status of 'completed,' 'discontinued,' or 'cancelled' were included in our calculations. Discontinued & cancelled appointments are included since their exclusion may bias estimates of wait times downwards (e.g. if a Veteran is unsatisfied with the wait and thus cancels their appointment). Incomplete consult requests were excluded.

The Con.Consult table in the CDW may be linked to other tables to identify consult type (e.g. cardiology, gastroenterology). The Con.ConsultActivity table tracks changes to the status of a consult – e.g. there are separate rows for when a consult is created, approved, scheduled, completed, cancelled, or discontinued. We use the 'ActivityDateTime' field to calculate three outcome measures:

1) Days to Approved, a measure of the difference between dates for when a consult is created and when it has been approved by the local VHA medical center. For community care, this is when the veteran was authorized to seek care in the community.
2) Days to Scheduled, a measure of the difference between when a consult is approved and when the appointment is scheduled. Note that for community care, this measure represents the date the VHA learned the veteran scheduled an appointment.
3) Days to Completed, a measure of the difference between when a consult is approved and when the initial appointment was completed.

We stratified these outcomes by VHA medical center, VHA/CC, and specialty. There a couple important caveats to these measures:

1) For community care, a consult activity of "SCHEDULED" is when the local CC office follows up with a Veteran and finds out they have made the appointment; this is likely not the same day the Veteran actually made the appointment.
2) For community care, a consult activity of "COMPLETED" is when all documentation was received by the CC provider; this is not the same as the actual appointment date.

There is no remedy for #1; thus we excluded this outcome from our results. For #2, the Con.Consult and Con.ConsultActivity tables are also linked to the Appt.Appointments table through a unique ConsultSID, which allows us to observe actual appointment dates. These appointment dates were validated against medical records and notes in the VHA Computerized Patient Record System (CPRS).

The number of non-VHA consults that we can identify increased sharply starting in 2018 (see Appendix Figure). This coincides with official VHA guidance on how to record these consults in the data (specifically the introduction of stop code '669' and inclusion of 'COMMUNITY CARE' in the 'ToRequestServiceName' field of the Con.Consult CDW table). We estimate that our code may only capture 1/4 to 1/2 of non-VHA consults as prior to 2018; the remainder are erroneously counted as VHA consults due to the lack of standard labelling procedures across VHA medical centers.

The implementation of stop code '669' has enabled better identification of community care consults. Unfortunately, this is a generalist stop code that does indicate consult specialty. We follow a

© 2020 Griffith KN et al. *JAMA Network Open.*

tiered approach to try and identify the specialties of these 669 stop codes through 1) linkage to other CDW tables; and 2) text matching on specialty names based on the 'ToRequestServiceName' field of the Con.Consult CDW table. In our tests, 87% of stop codes are matched to more informative stop codes. The remaining 13% were dropped from our analyses.

Lastly, we exclude what we call 'zero day' consults from our analysis. Some consults are opened and closed immediately, or within a matter of minutes/hours. A chart review using CPRS indicated these are mostly e-consults, text/email messages between providers, and not actual in-person appointments. Leveraging the 'ToRequestServiceName' field, we identify consult types that had an average completion time of <= 0.2 days during 2008-2018 an excluded those from our analysis.

Appendix Figure. Number of stations reporting CC/VHA consults and consult volume over time

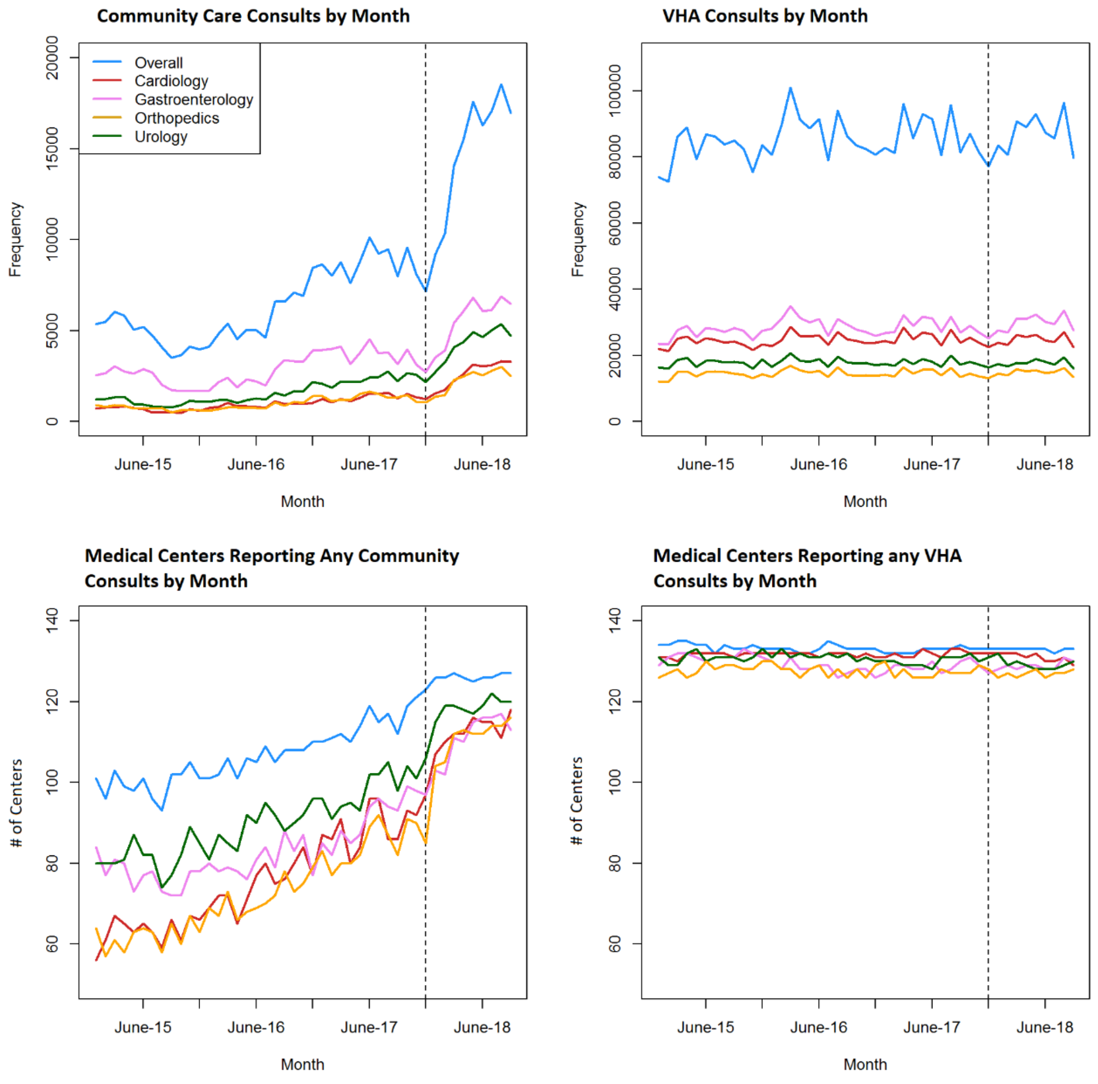

© 2020 Griffith KN et al. *JAMA Network Open.*
